# Clinical Profiles of Children With Sickle Cell Anaemia Presenting With Acute Clinical Events: A Single-Center Study

**DOI:** 10.7759/cureus.39008

**Published:** 2023-05-14

**Authors:** Anwesha Singh, Chandrakant Bokade, Bhagyashree Tirpude, Milind M Suryawanshi, Lakshmikant A Rohadkar

**Affiliations:** 1 Pediatrics and Neonatology, Indira Gandhi Government Medical College and Hospital, Nagpur, IND

**Keywords:** hydroxyurea, hemolytic crisis, hb electrophoresis, high-performance liquid chromatography (hplc), sickle cell disease (scd)

## Abstract

Background: Sickle cell disease is a common genetic disorder characterised by chronic haemolytic anaemia and vaso-occlusive crisis. Sickle cell anaemia (SCA) has both short-term effects in the form of acute clinical events and long-term repercussions seen with chronic multiorgan involvement. It is associated with significant morbidity and mortality. In India, the disease is largely undocumented. Thus, there is an urgent need to highlight the features of the disease so that locally appropriate models of care may be implemented.

Objective: This study aims to evaluate acute clinical events in SCA and to provide data that may help to reduce the rate of morbidity and mortality associated with this disease by early interventions.

Materials and methods: A cross-sectional observational study was conducted between November 2020 and May 2022 at Indira Gandhi Government Medical College and Hospital, Nagpur, Central India. The inclusion criteria included previously diagnosed patients of SCA (homozygous sickle cell disease) on high-performance liquid chromatography (HPLC) between the age groups of six months and 12 years, presenting with acute clinical events. The exclusion criteria included patients younger than six months and older than 12 years of age, and all patients with other haemoglobinopathies and sickle cell trait. The study was approved by the Institutional Ethical Committee. All the data was entered into a well-designed Microsoft Office Excel spreadsheet (v 2019, Microsoft, Washington, USA). All the clinical, biochemical, and haematological data were tabulated and analysed.

Results: A total of 100 children with sickle cell disease diagnosed by HPLC were enrolled during the study period. About 215 acute clinical events among the 100 cases were recorded, for which they were admitted to the paediatric ward or PICU. The majority (35%, n=35) were seen in the age group of six to nine years (school-going age). About 52% were male and 48% were female (male-to-female ratio= 1.08:1). Pain was the most common symptom. The highest incidence of 36.75% (n=79) was seen with acute painful crises and was the most common indication of hospitalisation, followed by acute febrile illness (AFI) (34.42%, n=74), aplastic crisis (10.23%, n=22), splenic sequestration crisis (9.77%, n=21), hepatobiliary involvement (3.72%, n=8), acute chest syndrome and haemolytic crisis (each 1.86%, n=4), and stroke (1.40%, n=3). In cases of having foetal haemoglobin (HbF) ≥20%, the incidence of acute painful crisis (p=0.0001), hand-foot syndrome (p=0.047), aplastic crisis (p=0.033), splenic sequestration crisis (p=0.039), and AFI (p=0.035) was low as compared to cases having HbF ≤20% which was statistically significant. The incidence of acute painful crisis, hand-foot syndrome, and an aplastic crisis was significantly low in patients receiving hydroxyurea therapy as compared to patients who were not on hydroxyurea. Out of 100 cases, four died during the study period, three died because of splenic sequestration crisis with septic shock, and one died due to hepatic encephalopathy due to haemolytic crisis with septic shock.

Conclusion: Acute clinical events in sickle cell disease can have significant morbidity and mortality in the paediatric age group. The nutritional status of sickle cell disease children must be given due importance. Early initiation of hydroxyurea must be encouraged to maintain higher HbF levels, which plays a significant role in reducing morbidity.

## Introduction

Sickle cell disease is an inherited blood condition that is most common among people of African, Arabian, and Indian origin [1] that is caused by a single nucleotide mutation that substitutes glutamic acid for valine in the sixth position of the β-globin gene [2]. During hypoxic conditions, the red blood cell becomes sickled, and the resulting change in structure restricts circulation causing obstruction of the blood flow within the capillaries and early destruction of the cell [3].

The awareness about sickle cell disease is highly restricted and is generally diagnosed after the incidence of one of its many complications. Many times death occurs before the disease can be diagnosed, and parents do not have a notion about the life-threatening crises that their child may suffer from. Hence, it has become immensely important to educate the masses to further reduce the mortality and morbidity associated with sickle cell disease.

A definitive cure is not currently available for patients with sickle cell anaemia (SCA). Existing therapies are only focused on symptom management and do not alter the natural history of the disease. These therapies are comprised of hydration, prevention of infections, pain management, proper nutrition, and precautions against adverse weather conditions. Hydroxyurea is an FDA-approved drug used in the management of sickle cell disease and has been found to be an effective therapy, as it reduces the number of hospitalisations, decreases mortality, and improves the quality of life [4].

Bone marrow transplantation is curative for SCA, but there is a lack of donor availability, as well as long-term adverse effects of a bone marrow transplant. In India, the disease is largely undocumented. Thus, there is an urgent need to throw light upon the features of disease so that locally appropriate models of care may be evolved [5].

The current scenario calls for a widespread screening programme to identify the disease at an early stage. The following study is designed with the aim to evaluate acute clinical events in sickle cell disease and to provide data that will help to reduce the rate of morbidity and mortality associated with this disease by early interventions.

## Materials and methods

Study design and setting

A cross-sectional observational healthcare facility-based study was conducted in the Department of Pediatrics at a tertiary care hospital in Nagpur, Central India, between November 2020 and May 2022 (18 months).

Study participants

The inclusion criteria included children between the age group of six months and 12 years. A sample size of 100 subjects, consisting of previously diagnosed cases of SCA (SS pattern), was included in the study. The sample size was calculated using a previous study by Patel et al. [6], which observed that out of 47 patients with sickle cell disease, 59.57% had an acute painful crisis, 25.53% had an acute febrile illness (AFI), 2.12% had acute chest syndrome, and 46.80% had severe anaemia. Taking this value as a reference, the minimum required sample size with a 10% margin of error and a 5% level of significance is 97 patients. To reduce the margin of error, the total sample size taken is 100.

The formula used is N ≥ (p (1 -p))/(ME/zα)2

where,

Zα is the value of Z at a two-sided alpha error of 5%, ME is the margin of error, and p is the percentage of various acute events.

Calculations:

1. Acute painful crisis n>=((.5957*(1-.5957))/(.1/1.96)2=92.52=93 (approx.)

2. AFI n>=((.2553*(1-.2553))/(.1/1.96)2=73.04=74 (approx.)

3. Acute chest syndrome n>=((.0212*(1-.0212))/(.1/1.96)2=7.97=8 (approx.)

4. Severe anaemia n>=((.4680*(1-.4680))/(.1/1.96)2=95.64=97 (approx.)

Inclusion Criteria for Cases

Previously diagnosed paediatric cases of SCA by high-performance liquid chromatography (HPLC) or Hb electrophoresis between six months and 12 years of age with any acute clinical event.

1. Paediatric cases of SCA with any acute clinical event

2. Patients between six months and 12 years of age

Exclusion Criteria for Cases

1. Cases of sickle cell trait

2. Cases of other haemoglobinopathies

3. Patients of less than six months and more than 12 years of age

Data collections

A detailed history of the demographic profile of the patient (age, sex, socio-economic status, and place of residence), nutritional status, immunisation status, and treatment with hydroxyurea was recorded. Clinical presentation of the patient including AFI, aplastic crisis, acute painful events, hand-foot syndrome, splenic sequestration crisis, acute chest syndrome, acute central nervous system events, and acute haemolytic crisis at the time of admission and detailed physical examination findings were recorded in the predesigned case report proforma.

The laboratory investigations including haemoglobin levels, foetal haemoglobin (HbF) levels, and various other relevant biochemical (liver function, kidney function, C-reactive protein, etc.), radiological (local ultrasonography, local X-ray imaging, and MRI, etc.), and microbiological (serological assays, urinalysis and culture, blood cultures, etc.) investigations depending on the clinical presentation of the patient were done. The patients admitted were treated as per the standard institutional treatment protocol.

Statistics analysis

The presentation of the categorical variables was done in the form of numbers and percentages (%). On the other hand, the quantitative data were presented as the means ± SD. A comparison of the incidence rate of different events was done. The data entry was done in the Microsoft Office Excel spreadsheet (v 2019, Microsoft, Washington, USA), and the final analysis was done with the use of SPSS Statistics version 25.0 (IBM Corp. Released 2017. IBM SPSS Statistics for Windows, Version 25.0. Armonk, NY: IBM Corp.). For statistical significance, a p-value of less than 0.05 was considered statistically significant.

Ethical consideration

Ethical clearance for conducting the study was obtained from the Institutional Ethics Committee. Information obtained during the study is confidential. Written informed consent in the local language was obtained from the parents or guardians of each enrolled patient who were willing to get enrolled in the study after explaining to them the nature of the study.

## Results

A total of 100 cases of homozygous sickle cell disease (SS pattern) between the age group of six months and 12 years who were admitted to the paediatrics ward and PICU with acute clinical events were enrolled. Out of the total 100 cases of SCA enrolled in the study, 52% (n=52) were males and 48% (n=48) were females.

As shown in Table 1, out of 100 cases of SCA, 35 (35%) were in the age group of between six and nine years, 30 (30%) were between nine and 12 years, 23 (23%) were between three and six years, and 12 (12%) were in the age group of six months and three years. Out of the 100 cases enrolled, 56% (n=56) were malnourished. Among the 56 cases that had evidence of malnutrition, 25% (n=25) were below five years of age and 31% (n=31) were older than five years.

**Table 1:** Age-wise distribution of case

Age group	Cases (n=100) no.	%
6 month-3 years	12	12
3 years- 6 years	23	23
6 years- 9 years	35	35
9 years- 12 years	30	30
Total	100	100

Table 2 shows the overall distribution of acute clinical events among all the cases enrolled in the study. A total of 215 acute morbid events were recorded among the 100 cases of the study. The highest incidence of 36.75% (n=79) was seen with acute painful crises. Out of this, 9.30% (n=20) events were those of hand-foot syndrome, and 27.45% (n=59) were of other painful crises. AFI had the second highest incidence of 34.42% (n=74), followed by 10.22% (n=22) of aplastic crisis, 9.77% (n=21) of splenic sequestration crises, 3.72% (n=8) hepatobiliary involvement, 1.86% (n=4) of acute chest syndrome, 1.86% (n=4) of haemolytic crises, and 1.40% (n=3) of stroke.

**Table 2:** Distribution of acute clinical events indicated for hospitalisation AFI: acute febrile illness

Acute clinical event		Cases = 100; acute events = 215)	
		No.	%
Acute painful crisis		79	36.75
	(i) Hand-foot syndrome	20	9.30
	(ii) Others	59	27.45
Acute chest syndrome		04	1.86
Aplastic crisis		22	10.23
Splenic sequestration crisis		21	9.77
AFI		74	34.42
Stroke		03	1.40
Haemolytic crisis		04	1.86
Hepatobiliary involvement		08	3.72
Total acute events		215	100

Out of the total 74 events of AFI, in 66.23% (n=49) of cases, organisms were isolated on blood culture, and the most common organism isolated was Salmonella typhi.

As shown in Table 3, The incidence of acute painful crisis in cases having HbF ≤20% was 94.94% (n=75), while that in cases having HbF ≥20% was 5.06% (n=4), which was found to be statistically significant (p=0.0001). The incidence of hand-foot syndrome in cases having HbF ≤20% was 95% (n=19), while that in cases having HbF ≥20% was 5% (n=1), which was found to be statistically significant (p=0.047). The incidence of aplastic crisis in cases having HbF ≤20% was 95.46% (n=21), while that in cases having HbF ≥20% was 4.55% (n=1), which was found to be statistically significant (p=0.033). The incidence of splenic sequestration crisis in cases having HbF ≤20% was 95.24% (n=20), while that in cases having HbF ≥20% was 4.76% (n=1), which was found to be statistically significant (p=0.039). The incidence of AFI in cases having HbF ≤20% was 75% (n=3), while that in cases having HbF ≥20% was 25% (n=1), but was not found to be statistically significant (p=0.035).

**Table 3:** Correlation of HbF levels with acute clinical events of SCA AFI: acute febrile illness

		HbF percentage			
Acute clinical event		<20% (cases =76)	>20% (cases =24)	Total (cases =100)	p-value
Acute painful crisis		75	04	79	0.0001
	(i) Hand-foot syndrome	19	01	20	0.047
	ii) Others	56	03	59	0.0007
Acute chest syndrome		03	01	04	0.963
Aplastic crisis		21	01	22	0.033
Splenic sequestration crisis		20	01	21	0.039
AFI		60	14	74	0.035
Stroke		02	01	03	0.705
Haemolytic crisis		03	01	04	0.963
Hepatobiliary involvement		06	02	08	0.947
Total acute events		190	25	215	

As shown in Table 4, the incidence of acute painful crisis in the cases who were on hydroxyurea was 3.39% (n=2) and was 96.61% (n=57) in those who were not on hydroxyurea, and this was found to be statistically significant (p=0.0008). The incidence of hand-foot syndrome in the cases who were on hydroxyurea was 5% (n=1) and was 95% (n=19) in those who were not on hydroxyurea, and this was found to be statistically significant (p=0.015). The incidence of aplastic crisis in the cases who were on hydroxyurea was 9.09% (n=2) and was 90.91% (n=22) in those who were not on hydroxyurea, and this was found to be statistically significant (p=0.032).

**Table 4:** Correlation between the incidence of acute clinical events and hydroxyurea therapy AFI: acute febrile illness

		Hydroxyurea therapy			
Acute clinical event		Received (n =30)	Not received (n =70)	Total (n=100)	p-value
Acute painful crisis		03	76	79	0.0291
	(i) Hand-foot syndrome	01	19	20	0.015
	(ii) Others	02	57	59	0.008
Acute chest syndrome		02	02	04	0.383
Aplastic crisis		02	20	22	0.032
Splenic sequestration crisis		07	14	21	0.739
AFI		20	54	74	0.577
Stroke		00	03	03	0.257
Haemolytic crisis		01	03	04	0.827
Hepatobiliary involvement		03	05	08	0.643
Total acute events		38	177	215	

## Discussion

Sickle cell disease is the most common heritable haemoglobinopathy worldwide, with multisystem involvement. SCA has both short-term effects in the form of acute clinical events and long-term repercussions seen with chronic multiorgan involvement. It also affects the long-term physical and neuro-cognitive growth of the patients [7].

Acute clinical events affect the day-to-day living of children, including the execution of routine activities, school attendance, and play activities [8]. It is, therefore, necessary to have a piece of knowledge about the profile of acute clinical events that increase morbidity among patients with SCA so that appropriate interventions can be taken at the right time.

In this study, 100 children with sickle cell disease with SS patterns between the age group of six months and 12 years were enrolled. Out of the total patients, 52% were male, and 48% were females (male-to-female ratio= 1.08:1). In a study conducted by Salman [7], 91 (56.88%) were male, and 69 (43.12%) were female with ages ranging from 0 to 14 years. Similar results were found in studies conducted by Patel et al., Jain et al. [8], Lyra et al. [9], Nayak et al. [10], Abideen et al. [11], and Faruk et al. [12].

It was observed that the highest number of cases, that is, 35% (n=35), were seen in the age group of six years to nine years (school-going age), followed by 30% (n=30) cases in nine years to 12 years (pre-adolescent age), 23% (n=23) in three years to six years (preschool age), and 12% (n=12) in six months to three years.

In the study done by Patel et al., most patients belonged to the age group of 5-12 years, comprising 68.08% of total sickle cell disease patients. In the study done by Jain et al., infants constituted 18% of the study population, while 56% were younger than three years of age.

The probable reason behind the highest incidence of acute events in the school-going and pre-adolescent age group may be greater communication and cross-infection from their healthier counterparts. Moreover, as the child grows older, and parents become more informed about the disease, they tend to have more hospital visits for the same [1].

Out of the 100 cases enrolled, 44% (n=44) had normal nutrition, while 56% (n=56) were malnourished. In the study done by Patel et al., 63.82% of SCA patients had undernutrition. Growth delay starts in early childhood but becomes more apparent during adolescence. Apparently, adequate nutritional supplement at a young age, thus, is a prerequisite. This emphasises the role of a diet rich in proteins and adequate calories, along with nutritional supplements such as folic acid and multivitamins in children with SCA.

A total of 215 acute morbid events were recorded among the 100 cases of the study. The highest incidence of 36.75% (n=79) was seen with acute painful crises and was the most common indication of hospitalisation, followed by AFI (34.42%, n=74), aplastic crisis (10.23%, n=22), splenic sequestration crisis (9.77%, n=21), and hepatobiliary involvement (3.72%,n=8). Similar results were found in a study done by Patel et al. Acute painful crisis (59.01%) was the most common cause of hospitalization, followed by severe anaemia (39.34%) and infections (36.06%).

In the study done by Jain et al., hospitalisation with AFI (31%) was the most common morbid event, followed by severe anaemia (30%) and acute painful events (20%). Morbid events like priapism, leg ulcers, and avascular osteonecrosis were not seen. In the study done by Kamble et al. [13], the vaso-occlusive crisis was the most common cause of hospitalisation. In the study done by Lyra et al., vaso-occlusion followed by AFI was the most common cause of hospitalisation. In the study done by Salman, Acute painful crisis was the most common cause of hospitalisation events (73.84%), followed by infection (9.28%), ACS (8.02%), and acute splenic sequestration crisis in 6.32%.

It was observed that HbF levels affected the incidence of some acute clinical events. In cases having HbF ≥20%, the incidence of acute painful crisis, hand-foot syndrome, aplastic crisis, splenic sequestration crisis, and AFI was low as compared to cases having HbF ≤20% which was statistically significant. A similar observation was found in the study done by Jain et al. Forty-five patients had HbF of more than 20% (26.80±4.81%), while 40 patients had HbF of less than 20% (14.36 ± 3.96%). Total morbid events, episodes of severe anaemia, acute painful events, and the hand-foot syndrome were significantly more common in those with lesser HbF at baseline.

The correlation between the incidence of acute clinical events and the use of hydroxyurea was analysed. The incidence of acute painful crisis, hand-foot syndrome, and an aplastic crisis was significantly low in patients receiving hydroxyurea therapy as compared to patients who were not on hydroxyurea. Similar results were seen in a study done by Ofakunrin et al. [14].

The number of subjects who had more than two episodes of painful crises reduced from 27 (50%) to 2 (2.7%) (p < 0.001), while those who had acute chest syndrome reduced from 6 (11.1%) to 0 (0.0%; p < 0.001). The risk of being transfused more than once was 0.11 times the risk in the 12-month period preceding therapy (95% CI = 0.02-0.85; p = 0.016). Similarly, the risk of hospital stay >7 days was 0.08 times the risk at the baseline (95% CI = 0.02-0.24; p < 0.0001). A significant reduction in acute painful events and blood transfusion rate was also observed in studies performed by Kenney et al. [15], Youssry et al. [16], Patel et al. [17], and Dipty et al. [18].

Out of the total number of cases enrolled, 48% (n=48) received a blood transfusion, while the remaining 52% (n=52) did not receive a blood transfusion. The indications of blood transfusion among the different cases of SCA were 95.45% (n=21), 90.47% (n=19) given for splenic sequestration crisis, 75% (n=3) for ACS, 75% (n=3) for a haemolytic crisis, and 2.07% (n=2) for AFI with anaemia.

The outcome of the study was classified into discharged, discharged against medical advice (DAMA), or death. Out of the 100 children who were enrolled in the study, 88% (n=88) were discharged after completion of treatment from a hospital, 8% (n=8) took DAMA, and 4% (n=4) died during the study period. Out of the four children who succumbed, 75% (n=3) died because of splenic sequestration crisis with sepsis with septic shock, and 25% (n=1) died due to hepatic encephalopathy due to haemolytic crisis with sepsis with septic shock. In the study done by Jain et al., two patients expired during the study period; one death was due to a splenic sequestration crisis and the other was due to severe sepsis.

This study has limitations. It is an observational study with a small sample size. The study only included patients with SC who needed hospitalisation. Lastly, the study did not consider outpatient burdens who do not visit hospitals.

## Conclusions

Our study shows that acute clinical events in sickle cell disease can have significant morbidity and mortality in the paediatric age group. It is necessary to identify the acute morbid events at an early stage so that appropriate interventions can be made. The nutritional status of children with sickle cell disease must be given due importance. Early initiation of hydroxyurea must be encouraged to maintain higher HbF levels, which plays a significant role in reducing morbidity. Education and awareness among parents with sickle cell disease must be empowered.
